# Coupled Temperature–Flow Field and Microstructure Numerical Simulation of the Solidification Process for Cu-3Ti-0.2Fe Alloy

**DOI:** 10.3390/ma18112478

**Published:** 2025-05-25

**Authors:** Jiangwei Hu, Qingjuan Wang, Kuaishe Wang, Wen Wang, Fengming Qiang, Longxin Li

**Affiliations:** School of Metallurgical Engineering, Xi’an University of Architecture and Technology, Xi’an 710055, China; 18592033147@163.com (J.H.); wangkuaishe888@126.com (K.W.); wangwen2025@126.com (W.W.); lilongxin0208@163.com (L.L.)

**Keywords:** Cu-3Ti-0.2Fe alloy, temperature field, flow field, solidification microstructure

## Abstract

This work investigates the time-dependent changes in temperature, flow, and solidification microstructure under various cooling conditions. The mechanism of the effects of different pouring temperatures on the morphology and evolution of the solidification microstructure is explored. During gradual cooling, the temperature distribution remained consistent and the solid–liquid interface extended to its furthest extent. In contrast, water cooling generated the most pronounced temperature gradient at the solidification front, which was conducive to the development of columnar grains. Specifically, the maximum solidification rates at the center of the casting under the water-cooled copper mold, copper mold, and ceramic mold conditions were 2.71 mm/s, 1.45 mm/s, and 0.95 mm/s, respectively, with water cooling achieving the fastest rate. In the early stages of solidification, the flow velocity at the casting center was relatively high, and during slow cooling, the molten material tended to flow toward the surface. When air cooling was applied, the molten material at the center migrated outward, while under water cooling, the fluid moved in an upward direction. At a heat transfer coefficient of 100 W/(m^2^·K), the alloy primarily formed equiaxed grains; however, at 5000 W/(m^2^·K), the proportion of columnar grains increased significantly, and the average grain area expanded from 3.664 × 10^−6^ m^2^ to 4.441 × 10^−6^ m^2^. Additionally, as the pouring temperature increased from 1100 °C to 1200 °C, the number of grains decreased, while the average radius grew from 1.665 × 10^−3^ m to 1.820 × 10^−3^ m, resulting in a reduced fraction of equiaxed grains. This study provides valuable theoretical insights for optimizing the solidification process of this particular alloy.

## 1. Introduction

High-performance elastic copper-based alloys primarily consist of the Cu-Be family [1], Cu-Sn group [2], and Cu-Ti category [3]. These materials are extensively employed in electrical and electronic applications, including wiring systems, circuitry, optical fiber cables, conductive springs, and contact connectors, owing to their exceptional mechanical strength, superior thermal conductivity, and outstanding electrical properties [4]. Nevertheless, Cu-Be alloys face limitations due to their inadequate resistance to stress relaxation at elevated temperatures, along with the significant health hazards posed by beryllium, a highly toxic and carcinogenic element [5]. As a result, their usage is increasingly being regulated or phased out. In contrast, Cu-Ti alloys have garnered considerable interest because of their remarkable strength, superior elastic properties, excellent weldability, and strong fatigue resistance. Additionally, Cu-Ti alloys have abundant raw material reserves, do not produce toxic substances during production, and are cost-effective, making them the most promising substitute for Cu-Be alloys [6,7,8].

With the rapid development of the fields in which they show application potential, Cu-Ti alloys are expected to meet higher performance requirements. To enhance the comprehensive properties of Cu-Ti alloys, researchers have explored the incorporation of ternary alloying elements, including Mg [9], B [10], and Fe [11]. Zhao et al. [12] examined the influence of minor Fe and Cr additions on the microstructural characteristics and mechanical behavior of Cu-3Ti alloys. Their findings revealed that introducing trace Fe during solid-solution treatment led to the formation of Fe_2_Ti intermetallic phases, which contributed to grain size reduction. Similarly, Huang et al. [13] analyzed the role of dilute Fe additions in ultra-high-strength Cu-Ti alloys, focusing on microstructure development and performance metrics. Their study demonstrated that Fe microalloying enhanced elevated-temperature mechanical properties, ductility, and oxidation resistance. Collectively, these investigations confirm that minor Fe doping positively influences the workability of Cu-Ti alloys while preserving their high tensile strength under thermal exposure.

The Cellular Automaton–Finite Element (CAFE) model, combined with the Gaussian distribution nucleation model and the KGT model, can be used to simulate grain growth and microstructural transformations in castings. Rappaz et al. [14] first applied this method to the microstructure simulation of aluminum–silicon alloy solidification. Subsequently, Nastac [15] and Stefanescu [16] extended this method, advancing the application of CAFE in material design, process optimization, and performance prediction. In recent years, many researchers have made significant progress in grain size simulation studies using the CAFE method. Fang [17] employed the three-dimensional Cellular Automaton–Finite Element (3D-CAFE) approach to model the microstructure development of the Ag-28Cu-1Ni alloy in continuous casting. Their research systematically investigated how process variables, including nucleation undercooling, the casting height, the pouring temperature, the heat transfer coefficient, and the withdrawal rate, influenced the alloy’s solidified grain structure. Similarly, Liu et al. [18] utilized the CAFE technique to analyze grain formation in large-scale titanium slabs during electron beam cold hearth melting (EBCHM). Their study determined the optimal nucleation parameters for titanium ingot microstructure simulation, with a particular emphasis on the effects of the pouring temperature and withdrawal rate on the grain morphology. In a related work, Jing et al. [19] developed a 3D CAFE numerical model to predict the solidification behavior of the TC4 titanium alloy during vacuum arc remelting (VAR). Their simulations evaluated critical nucleation parameters, such as maximum bulk undercooling, nucleation site density, and nucleation undercooling distribution, and their role in governing the final grain structure.

The microstructures formed under different cooling conditions have a significant impact on the mechanical and functional properties of materials. The cooling rate, temperature gradient, and cooling method all affect the size of the grains, the composition of phases, and the morphology of the microstructure, thereby altering the mechanical behavior of the material, such as its strength, hardness, and toughness. Brittney Terry et al. [20] studied the effects of different cooling rates and Fe contents on the microstructures and mechanical properties of (CoCrCuTi) 100−xFex multi-entropy alloys. The results showed that a higher cooling rate promoted the formation of uniform dendritic structures, thereby increasing the hardness and fracture toughness of the alloy. C. Crozet et al. [21] studied the effects of different cooling rates on γ→α phase transformation and metastable states in Fe-Cu alloys. The results showed that the cooling rate had a significant influence on the phase transformation process and the formation of metastable phases. Therefore, by comparing and analyzing the final microstructures formed under different cooling conditions, we can elucidate the regulatory role of the cooling process on material properties, providing a theoretical basis and technical support for material design and optimization.

Although significant progress has been made in the simulation of alloy solidification microstructures using the CAFE model [22,23,24,25,26], this study still implemented unique innovations. Previous studies mainly focused on aluminum–silicon alloys, Ag-28Cu-1Ni alloys, and titanium alloys. Systematic simulation studies on the solidification behavior of Cu-Ti-Fe ternary alloys are relatively scarce in the literature. This study is the first to include a comprehensive numerical simulation of the solidification process of the Cu-3Ti-0.2Fe alloy with three different cooling modes and analyze, in detail, the changes in the thermal field, flow field, and solidification microstructure according to cooling conditions and pouring temperature. In this study, the unique mechanisms underlying the microstructure evolution of this alloy under different cooling and pouring conditions are revealed. These results provide a new theoretical basis for optimizing the solidification process of Cu-Ti-Fe-based alloys. Moreover, the Cu-3Ti-0.2Fe alloy is a high-performance elastic copper-based alloy with potential application value, so studying its solidification process is of great practical significance in promoting its application in actual production. This aspect has not been covered or comprehensively explored in previous studies.

## 2. Model Establishment and Parameter Determination

### 2.1. Macroscopic Model

#### 2.1.1. Thermophysical Property Calculation

To evaluate thermal physical properties, including the dynamic viscosity, enthalpy, mass density, convective heat transfer coefficient, melting enthalpy, and liquid phase heat capacity, JMatPro software (version 13.2, Sente Software Company, Guildford, UK) is typically used. This software is widely recognized in the industry as a reliable tool because of its high accuracy and extensively verified calculation results [27]. This software is mainly based on a single-mixing model and a double-mixing model [28]:

(1) $$p=\sum_{i}x_{i}p_{i}+\sum_{i}\sum_{j\rangle i}x_{i}x_{j}\sum_{v}\Omega_{ij}^{v}{\left(x_{i}-x_{j}\right)}^{v}$$

where *P* denotes the phase’s thermophysical characteristics; *P_i_* corresponds to the pure constituent elements’ thermophysical parameters within said phase; *x_i_* and *x_j_* indicate the constituent elements’ molar concentrations in the phase; and *v* serves as the independent variable for establishing the binary interaction coefficient, $\Omega_{ij}^{v}$, expressed in mol L^−1^, with 0 ≤ *v* ≤ 2.

The thermomechanical parameters implemented in the ProCAST (version 2022) finite element simulation appear in Table 1.

#### 2.1.2. Governing Equations

Accounting for the three-dimensional unsteady flow behavior and thermal transport phenomena in molten metal, fundamental conservation equations were formulated based on mass, momentum, and energy principles. Additionally, taking gravity into account, a gravitational term was included in the momentum equation to make it applicable to both liquid and solid regions [15,29]. The governing equations used are as follows:

(1) Mass Conservation Equation.

(2) $$\frac{\partial \rho }{\partial t}+\frac{\partial \left(\rho u\right)}{\partial x}+\frac{\partial \left(\rho v\right)}{\partial y}+\frac{\partial \left(\rho w\right)}{\partial z}=0$$

(2) Momentum Conservation Equation.

(3) $$\begin{array}{l} \frac{\rho }{f_{1}}\frac{\partial u}{\partial t}+\frac{\rho }{f_{1}^{2}}\left(u\frac{\partial u}{\partial x}+v\frac{\partial v}{\partial y}+w\frac{\partial w}{\partial z}\right)=\\ -\frac{\partial p}{\partial x}+\rho g_{X}+\frac{\partial }{\partial x}\left(\frac{u}{f_{1}}\frac{\partial u}{\partial x}\right)+\frac{\partial }{\partial y}\left(\frac{u}{f_{1}}\frac{\partial u}{\partial y}\right)+\frac{\partial }{\partial z}\left(\frac{u}{f_{1}}\frac{\partial u}{\partial z}\right)-\frac{u^{2}}{k} \end{array}$$

(3) Energy Conservation Equation.

(4) $$\begin{array}{l} P\frac{\partial H}{\partial t}+P\frac{\partial H}{\partial t}\left(u\frac{\partial T}{\partial x}+v\frac{\partial T}{\partial y}+w\frac{\partial T}{\partial z}\right)=\\ \frac{\partial }{\partial x}\left(K_{T}\frac{\partial T}{\partial x}\right)+\frac{\partial }{\partial y}\left(K_{T}\frac{\partial T}{\partial y}\right)+\frac{\partial }{\partial z}\left(K_{T}\frac{\partial T}{\partial z}\right) \end{array}$$

where

(5) $$H\left(T\right)=\int_{0}^{T}C_{P}\left(T\right)dT+L\left(1-f_{s}\right)$$

where *u*, *v*, and *w* are velocity components in the *x*, *y*, and *z* directions, respectively, in m/s; *f*_1_ and *f*_2_ are the liquid and solid phase fractions, respectively; *P* denotes the pressure, in Pa; *g_x_* is the gravitational component in the *x* direction, in m/s^2^; *ρ* is the density, in kg/m^3^; *u* represents the permeability, in m^2^; *Cp* is the specific heat capacity, in J/(kg·°C); *H* is the enthalpy, in J/mol; and *L* represents the latent heat of fusion, in J/kg.

### 2.2. Microscopic Model

#### 2.2.1. Nucleation Model

**Figure 1:**
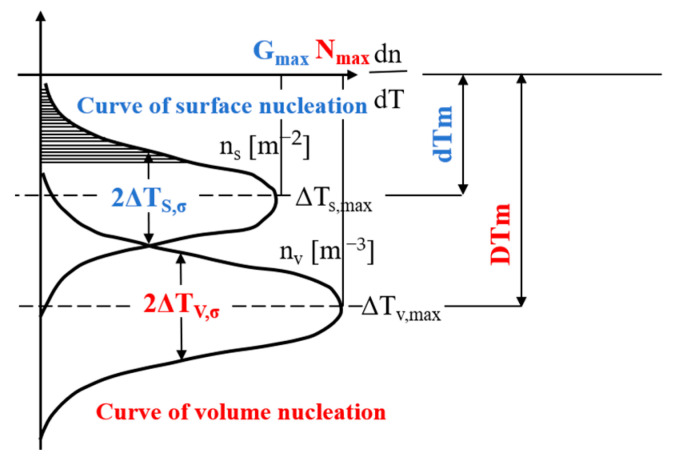
The Gaussian distribution function for the casting surface (indexed as ‘s’) and interior of the ingot (indexed as ‘v’).

During metal solidification, nucleation occurs through two distinct mechanisms: uniform nucleation and substrate-assisted nucleation. In a real solution environment, impurities and external surfaces (such as the container surface) are unavoidable. Except in specific laboratory conditions, homogeneous nucleation does not occur in liquid metals; thus, the primary solidification mechanism is heterogeneous nucleation. In simulating the microstructure of alloys, heterogeneous nucleation occurs mainly through two pathways: continuous nucleation and instantaneous nucleation. This research focused on progressive nucleation as the principal modeling approach, utilizing a Gaussian probability distribution to characterize the nucleation kinetics. The model’s conceptual framework is presented in Figure 1 [30].

(6) $$\frac{dn}{d\left(\Delta T\right)}=\frac{n_{\max}}{\sqrt{2\pi }\Delta T_{\sigma }}\exp\left[-\frac{{\left(\Delta T-\Delta T_{\max}\right)}^{2}}{2\Delta T_{\sigma }^{2}}\right]$$

where *n_max_* is the maximum nucleation density obtained by integrating the normal distribution from 0 to ∞, in m^−3^; Δ*T* denotes the undercooling, in K; Δ*T_max_* is the average nucleation undercooling, in K; and Δ*T_σ_* denotes the standard deviation of nucleation undercooling, in K.

The differential term dn quantifies the incremental change in grain density resulting from increased undercooling (Δ*T*). Under specified undercooling conditions, the resultant nuclei density *n*(Δ*T*) can be determined through the integration of the following distribution function:

(7) $$n\left(\Delta T\right)=\int_{0}^{\Delta T}\frac{\mathrm{dn}}{d\left(\Delta T\right)}d\Delta T$$

#### 2.2.2. Dendrite Tip Kinetic Growth Model

According to solidification principles, grain growth initiates when dendritic interfaces advance into undercooled molten metal. This growth process is governed by the synergistic effects of kinetic undercooling and solutal undercooling. The total undercooling (Δ*T*) at dendritic tips represents the cumulative contribution of four distinct undercooling components [31].

(8) $$\Delta T=\Delta T_{C}+\Delta T_{t}+T_{r}+T_{k}$$

where Δ*T_c_* represents compositional undercooling, in K; Δ*T_t_* denotes thermodynamic undercooling, in K; Δ*T_r_* represents solid–liquid interface curvature undercooling, in K; and Δ*T_k_* represents growth kinetic undercooling, in K.

In most metals, during normal solidification, the compositional undercooling is much greater than the other three types of undercooling. Therefore, the other three undercooling factors can be neglected, and the equation can be approximated as Δ*T* = Δ*T_c_*. Rappaz, Kurz, and others [8] simplified the relationship between v and Δ*T* in the KGT model. The commonly used computational formula in numerical simulations is as follows:

(9) $$v\left(\Delta T\right)=a_{2}\Delta T^{2}+a_{3}\Delta T^{3}$$

The coefficients a_2_ and a_3_ represent the kinetic parameters for the growth of the dendrite tip and are obtained through Equations (10) and (11). These parameters are derived from four fundamental material properties: the slope of the liquidus line (m_1_), the equilibrium distribution coefficient (*k_e_*), the liquidus diffusion rate (*D*_1_), and the Gibbs–Thomas coefficient (Γ).

(10) $$a_{2}=\frac{2k\Gamma \left(1-k\right)-\rho D^{2}}{2kmC_{0}\pi^{2}\Gamma {\left(1-k\right)}^{2}}$$

(11) $$a_{3}=\frac{D}{\pi \Gamma {\left(mC_{0}\right)}^{2}{\left(1-k\right)}^{2}}$$

The dendrite tip growth kinetic parameters are specified as *a*_2_ = 1.85 × 10^−7^/(sK^3^) and *a*_3_ = 2.02 × 10^−6^/(sK^3^).

### 2.3. Cellular Automaton–Finite Element Method Coupled Model

**Figure 2:**
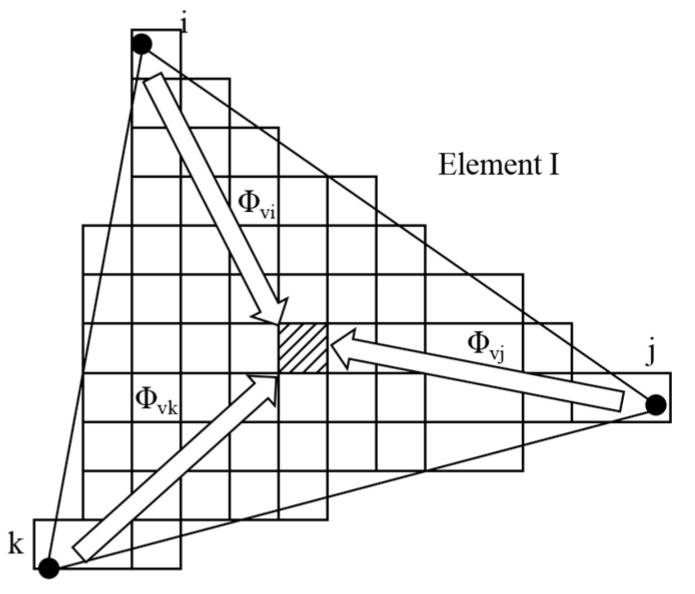
Relation between FE mesh and CA units [30].

The CAFE framework establishes transfer functions linking finite element discretization points with cellular automaton lattice sites, while phase transformation enthalpy from solidifying grains is accounted for in thermal computations to maintain microstructural–thermal field synchronization (Figure 2). For a given CA cell ν within the FE domain, the mapping weights Φ_νi_, Φ_νj_, and Φ_νk_ define its connectivity to the adjacent FE vertices i, j, and k. These coupling parameters enable temperature field reconstruction at FE coordinates through weighted interpolation, where dendritic evolution (nucleation→growth→coarsening) generates localized heat sources that dynamically modify nodal temperature distributions [30].

### 2.4. Modeling and Mesh Generation

**Figure 3:**
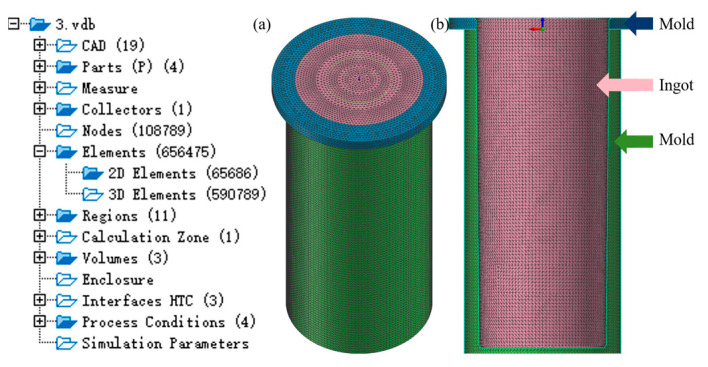
Model and mesh generation: (**a**) solid mold; (**b**) long section.

A 3D model of the mold was created using UG software (version NX 12.0, Siemens, Munich, Germany), and the constructed model was imported into ProCAST software (version 2022, *ESI*, Paris, France). The computational mesh was created using the MeshCAST component, employing a uniform element size of 3 mm (Figure 3). The generated discretization consisted of 108,789 nodal points, 65,686 surface elements, and 590,789 volumetric cells.

### 2.5. Experimental Procedure and Parameter Determination

#### 2.5.1. Experimental Procedure

In our experimental work, we utilized a 99.9 wt% purity electrolytic copper and titanium sponge, combined with commercial-grade iron, as a feedstock. Vacuum induction melting produced the target Cu-3Ti-0.2Fe (wt%) composition. The solidification modeling incorporated experimentally calibrated pouring temperatures, flow-adaptive water cooling parameters (h = f(v)), and full experimental condition replication. Phase transition temperatures were identified at *T_L_* = 1050 °C and *T_S_* = 880 °C.

Both the experimental and simulated castings shared identical dimensions (Ø115 mm × 210 mm). The ProCAST 3D-CAFE module simulated solidification dynamics (temperature/flow fields, microstructure) under three cooling regimes. To maintain experimental consistency, uniform interfacial heat transfer coefficients were applied. The cooling conditions varied by mold type, as follows. For the ceramic mold, the slow cooling (h = 100 W/(m^2^·K)) value was applicable to the production of precision instrument components, where uniformity of the organization is highly required, to improve the plasticity of the alloy and to eliminate internal stress. For the uncooled copper mold, the air cooling (h = 1000 W/(m^2^·K)) value was applicable for simulating conventional metal casting processes, such as the production of copper alloy valves and mechanical parts. The cooling rate was moderate, allowing for the formation of a mixed structure of columnar crystals and equiaxed crystals. For the water-cooled copper mold, the intensive quenching (h = 5000 W/(m^2^·K)) condition was in line with the actual production of high-strength and high-toughness alloys for aerospace applications. Through rapid cooling, a fine-grained structure was obtained, enhancing mechanical properties and thermal conductivity. Metallographic specimens were extracted 30 mm below the pouring cup. Cross-sections underwent mechanical polishing followed by etching (HCl:H_2_O:FeCl_3_ = 20 mL:50 mL:2.5 g) for microstructure characterization.

The elemental distribution of the Cu-Ti-Fe system under investigation is presented in Table 2, which is contrasted with the CuCrZr mold alloy composition provided in Table 3.

**Figure 4:**
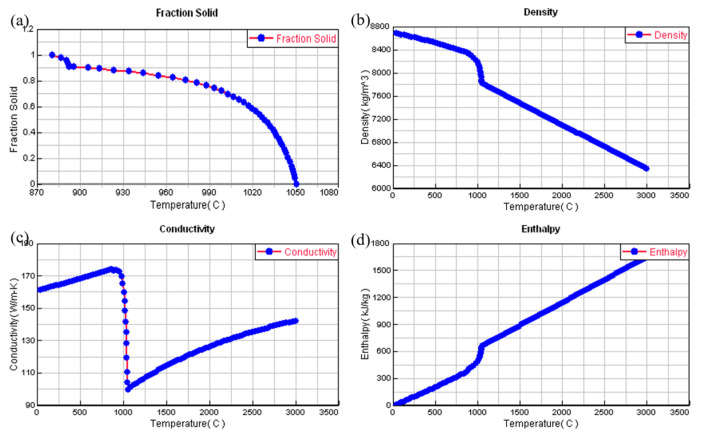
Illustration of the correlations between the thermophysical properties of the Cu-3Ti-0.2Fe alloy and temperature. (**a**) The correlation between the solid phase fraction and temperature, (**b**) the relationship between density and temperature, (**c**) the connection between thermal conductivity and temperature, and (**d**) the association between enthalpy and temperature.

The critical material characteristics considered in the numerical model included the phase fraction, mass density, heat conduction capacity, and thermal energy content, among others. These parameters were calculated using ProCAST software, as shown in Figure 4.

#### 2.5.2. Parameter Determination

**Figure 5:**
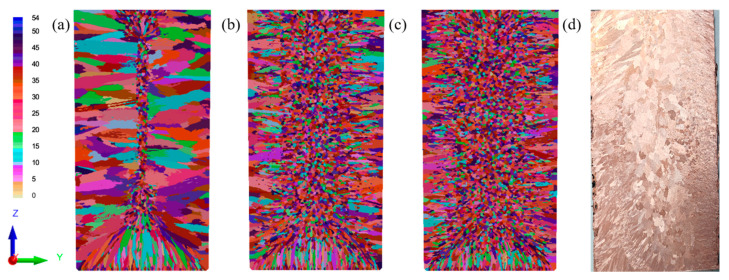
Simulation results of the Cu-3Ti-0.2Fe ingot under different average nucleation overcooling degrees (**a**) 1 K; (**b**) 2.5 K; (**c**) 5 K; (**d**) Experimental results.

In this study, the focus was on the average nucleation overcooling parameter, and it was assumed that the other two Gaussian parameters were specific empirical values. Table 4 lists the different Gaussian nucleation distribution parameters in the CAFE model. The average nucleation overcooling (Δ*T_v,max_*) is a single variable, while the other nucleation parameters are constants. Figure 5 shows the influence of the average overall overcooling on the microstructure of the Cu-3Ti-0.2Fe alloy, which determines the average nucleation overcooling in the molten metal as a whole.

The results show that when the average overcooling degree was 2.5 K, the simulation results were in good agreement with the actual experimental results. The grain morphology simulation results were relatively close to the experimental results. Based on this, the average overcooling degree of the Cu-3Ti-0.2Fe ingot was determined to be Δ*T_v,max_* = 2.5 K. This was used as the nucleation parameter in the prediction and analysis of the solidification structure evolution laws under different casting conditions, enhancing our ability to control the solidification structure of the Cu-3Ti-0.2Fe ingots by controlling the pouring temperature and cooling conditions.

ProCAST’s CAFE module simulated the alloy’s microstructure evolution, with key parameters, including the dendrite tip kinetics and nucleation characteristics (Table 5).

## 3. Results and Analysis

### 3.1. Temperature Field Analysis

**Figure 6:**
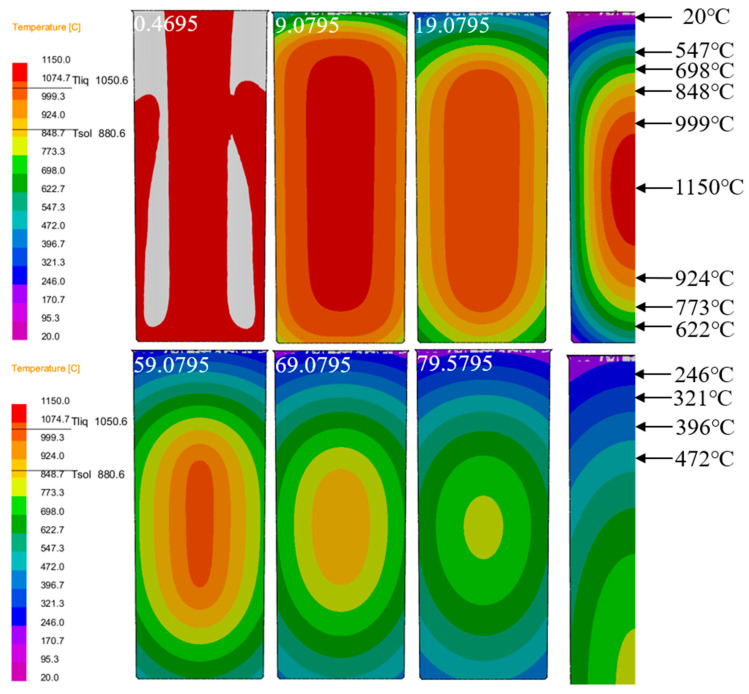
Temperature field distribution of the casting at different times under water cooling conditions for Cu-3Ti-0.2Fe.

For the temperature evolution analysis, Figure 6 displays the thermal profile evolution of the Cu-3Ti-0.2Fe casting during water-cooled solidification. The time-sequence images reveal progressive cooling throughout the process, demonstrating consistent temperature reduction as solidification progresses. At 0.4695 s, the pouring temperature of the casting reaches 1150 °C, depicted in red in the temperature field distribution map. As time progresses (from 9.0795 s to 79.5795 s), the color of the temperature field changes significantly, with the high-temperature regions (1150 °C, 999 °C, 924 °C, etc.) shrinking in size, while the low-temperature regions (20 °C, 246 °C, etc.) gradually expand. The casting solidifies from the outer surface inward. The two-dimensional heat extraction through the mold base initiates primary solidification at the bottom periphery, where molten metal first transitions to the solid phase. In the figure, it can be observed that the temperature at the edges of the mold bottom decreases first: the temperature starts high at 1150 °C and, as solidification progresses, drops to 773 °C, 622 °C, and so on. Later, solidification progresses radially inward from the mold walls, with localized temperatures decreasing systematically from the initial 999 °C readings. Finally, the molten metal in the center solidifies, with the temperature gradually decreasing, showing a clear temperature gradient. During phase transformation, a compositionally and thermally undercooled liquid region is sustained ahead of the growing solid phase. As the molten metal continues to solidify, heat transfer in the liquid phase increases, and the undercooled region expands. From the changes in the temperature field shown in the figure, the low-temperature range corresponding to the undercooled area increases, with initially fewer low-temperature regions, such as those at 20 °C and 246 °C, which gradually extend as solidification progresses. The degree of undercooling increases, thereby expanding the nucleation growth area. Starting from the relatively concentrated high-temperature regions, more low-temperature intervals appear, creating more favorable conditions for nucleation growth. Generally speaking, undercooling occurs first in the metal melt near the solid–liquid interface, providing the conditions for nucleation. Figure 6 reveals the progressive inward propagation of solid–liquid isotherms, accompanied by the continuous thickening of the solidified region. The temperature field visualization reveals that the isothermal surfaces move from high temperatures (1150 °C, 999 °C) toward lower temperatures, with the solidification region expanding from the edge inward. The temperature range of the solidification layer gradually shifts from higher-temperature to lower-temperature regions until the entire molten alloy has solidified. Furthermore, because of the symmetrical geometry and cooling conditions of the casting’s longitudinal section, the temperature field distribution becomes symmetric during solidification and cooling. Taking advantage of this symmetry, in this study, we examined only half of the horizontal cross-section, paying particular attention to the thermal and flow behaviors in the central zone.

**Figure 7:**
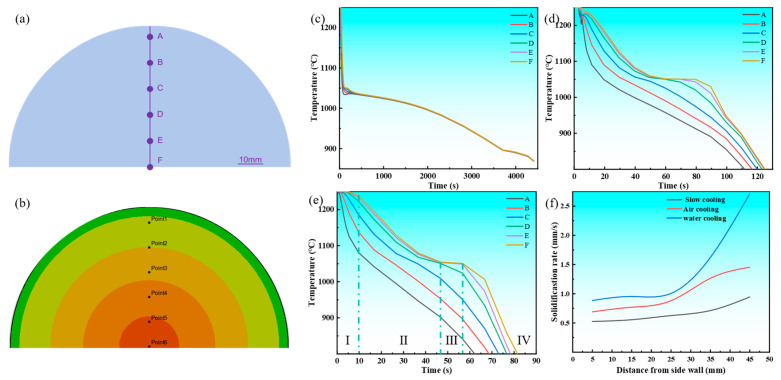
The distribution of copper-3% titanium-0.2% iron alloy at each measurement point, as well as the cooling and solidification rate curves at different positions. (**a**) Schematic diagram of the measurement points; (**b**) actual positions of the measurement points; (**c**) slow cooling; (**d**) air cooling; (**e**) water cooling; (**f**) solidification rate curve.

As depicted in Figure 7a, the arrangement of temperature monitoring points is clearly visible. The temperature at the center of the casting is set to 0, with measurement points placed every 10mm from the center to the surface, labeled sequentially as A–F. These measurement points provide a foundational reference for studying temperature variations at different locations. Figure 7c–f present the cooling and solidification rate curves of the Cu-3Ti-0.2Fe alloy under various cooling conditions. In all cases, the middle section of the cooling curve exhibits four distinct stages. The first stage, associated with mold filling, corresponds to the time interval between 0.4695 s and 9.0795 s (Figure 6). During this phase, molten metal is injected into the mold, and because of continuous liquid injection, the cooling curve does not show significant fluctuations. The second stage represents rapid solidification, initiated immediately after mold filling. Here, the significant temperature gradient between the molten metal and mold drives fast cooling until the temperature falls below the liquidus line, reaching this transition point at 19.0795 s (Figure 6). The third stage is the isothermal solidification stage, corresponding to 59.0795 s in Figure 6. During this stage, the cooling curve forms a stable flat platform, as the latent heat released during solidification exceeds the rate of heat loss. The fourth stage is the cooling stage, corresponding to 69.0795 s and 79.0795 s in Figure 6. Following complete solidification, the latent heat released during crystallization becomes insufficient to balance the heat transfer rate between the casting and mold, resulting in a steady temperature decrease. As evidenced in Figure 7c–f, under water cooling conditions, the latent heat release from the solidifying metal approximately offsets the casting’s heat loss during initial solidification. Under air cooling conditions, the temperature platform typically appears after some time has passed in the solidification process, while under slow cooling conditions, the platform appears in the later stages of solidification. Furthermore, at temperatures above the solidus, the divergence between cooling curves progressively narrows when comparing slow cooling, air cooling, and water cooling conditions. The findings demonstrate that slow cooling produces the most uniform temperature field distribution during solidification, with air cooling showing intermediate uniformity, while water cooling results in the least homogeneous thermal distribution.

The alloy’s solidification rate directly influences its microstructure refinement and consequent mechanical property enhancement. To quantify this relationship across different cooling conditions, we defined the solidification rate as the temperature gradient (M_i+1_ − M_i_) between adjacent measurement points divided by their corresponding liquidus attainment time difference (t_i+1_ − t_i_), where (M_i+1_ + M_i_)/2 represents the normalized distance from the mold sidewall.

Figure 7f demonstrates the relationship between the solidification rate and the distance from the mold sidewall for the Cu-3Ti-0.2Fe alloy under different cooling conditions. The key findings include the following: Distance Dependence: The solidification rate consistently increased with the distance from the sidewall for all cooling methods. Cooling Method Comparison: Water cooling achieved the highest maximum rate (2.71 mm/s), air cooling showed intermediate values (1.45 mm/s), and slow cooling yielded the lowest rates (0.95 mm/s). Rate Characteristics: Water cooling exhibited substantial rate fluctuations throughout solidification, slow cooling maintained stable rates (~0.67 mm/s at 40% completion), air cooling demonstrated a steadily increasing trend, and the water cooling rates surpassed the air cooling rates after 60% solidification. The most significant rate variations occurred in forced cooling conditions (air and water), with water cooling showing particularly pronounced fluctuations.

**Figure 8:**
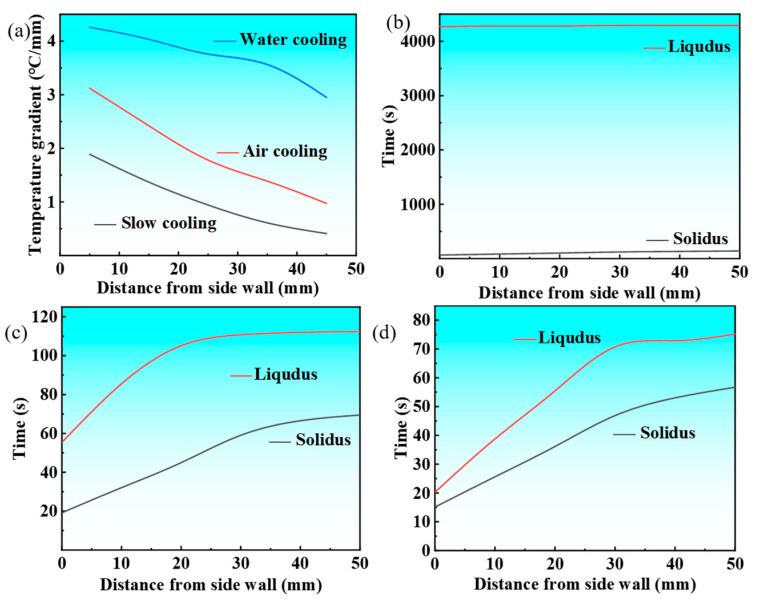
The temperature gradient at the solidification front and the width of the solid–liquid zone of the Cu-3Ti-0.2Fe alloy under diverse cooling conditions: (**a**) the temperature gradient; (**b**) slow cooling; (**c**) air cooling; (**d**) water cooling.

The temperature gradient at the solid–liquid interface plays a crucial role in solidification microstructure development, with higher gradients promoting preferential crystal growth and enhancing columnar grain formation, ultimately increasing their volume fraction in castings. To characterize the solidification front temperature gradient across various cooling conditions, the temperature difference between a specific measurement point X_i_ and the liquidus temperature, as well as the temperature difference between the adjacent point X_i+1_ and the liquidus temperature, is defined. This difference is then divided by the spatial distance between the two measurement points (M_i+1_ − M_i_) to characterize the temperature gradient at the solidification front. Figure 8 illustrates the relationship between the temperature gradient at the solidification front of the Cu-3Ti-0.2Fe alloy under different cooling conditions and its distance from the casting sidewall. Figure 8a demonstrates that cooling methods profoundly influence the solidification front temperature gradient. Water cooling produces the steepest gradient initially, due to its intense heat extraction capacity, though this gradient progressively declines as solidification advances, while remaining superior to those of air and slow cooling throughout the process. Air cooling generates intermediate gradients, while slow cooling maintains the most gradual and stable thermal profiles. Notably, both air and slow cooling exhibit stabilized gradients after reaching the 2/5 solidification stage. These thermal conditions directly affect microstructural development: the pronounced thermal gradients and non-uniform temperature distribution that occur with water cooling preferentially promote columnar grain formation through enhanced directional crystal growth along heat flux vectors, while the uniform thermal field observed with slow cooling yields more equiaxed structures. The observed gradient hierarchy (water > air > slow cooling) consistently correlates with increasing columnar grain dominance in the final microstructure.

The solid–liquid coexistence zone width, governed by solidus–liquidus spacing (Figure 8b–d), varies inversely with the temperature gradient. Slow cooling produces the widest zone, air cooling produces an intermediate zone, and water cooling produces the narrowest zones. Crucially, wider zones enhance equiaxed grain formation through crystal retention and increased nucleation sites. While slow cooling maintains stable widths, air cooling shows progressive widening. Water cooling exhibits a distinct two-stage behavior, i.e., initial contraction followed by rapid expansion, reflecting its unstable solidification kinetics. These cooling-dependent zone characteristics directly control the final grain morphology.

### 3.2. Flow Field Analysis

**Figure 9:**
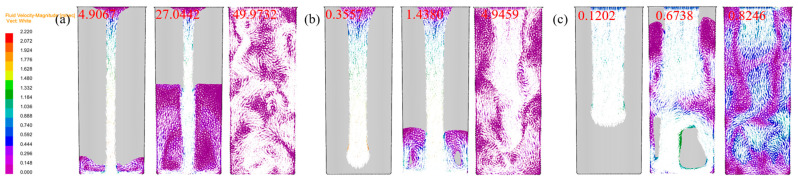
The flow tendencies of the Cu-3Ti-0.2Fe alloy at different times under various cooling conditions: (**a**) slow cooling; (**b**) air cooling; (**c**) water cooling.

During the solidification of the Cu-3Ti-0.2Fe alloy, density and temperature gradients induce buoyancy-driven convection within the melt. Figure 9 illustrates the evolving flow patterns under three cooling conditions. Initially, the highest flow velocities occur in the central region near the pouring gate, consistently exceeding surface velocities across all cooling methods. As solidification progresses, variations in shrinkage location and force direction cause distinct flow pattern divergences between cooling conditions.

Under slow cooling conditions, the flow field exhibits a symmetrical distribution along the centerline of the casting. With an air cooling time of 1.4380 s and a water cooling time of 0.6738 s, the flow field within the melt shows an uneven distribution, with a more chaotic flow direction. The increasing heat transfer coefficient enhances flow instability, with the water-cooled conditions particularly promoting vortical and turbulent melt behavior.

The melt exhibits distinct flow patterns under different cooling conditions post-solidification. Slow cooling induces inward-to-surface flow, while air cooling triggers outward melt migration from the center as rapid surface solidification occurs. In contrast, under water cooling conditions, the cooling conditions at the top surface fail to meet the full water cooling requirements, leading to a lower thermal conductivity in this region compared to other parts of the casting, which results in a slower heat dissipation rate. Therefore, as the solidification process nears completion, the fluid primarily moves toward the upper region.

**Figure 10:**
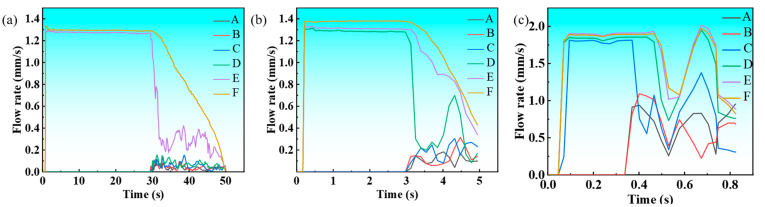
The flow velocity curves of Cu-3Ti-0.2Fe under different cooling conditions: (**a**) slow cooling; (**b**) air cooling; (**c**) water cooling.

Figure 10a–c present the temporal evolution of melt flow velocity under slow, air, and water cooling conditions, revealing consistent trends across all environments. Initially, density-driven buoyancy accelerates the flow velocity during early solidification. The velocity peaks at a characteristic point before declining as increasing alloy viscosity dominates at lower temperatures, ultimately ceasing flow. While all three conditions follow this general pattern, water cooling exhibits the highest peak velocity, followed by air and slow cooling, reflecting their respective heat extraction capacities. The velocity decay phase shows cooling-dependent characteristics: water cooling demonstrates the most abrupt decrease, while slow cooling maintains a more gradual deceleration, corresponding to their different solidification kinetics.

### 3.3. Solidification Microstructure

#### 3.3.1. Effect of Cooling Conditions on Solidification Microstructure

**Figure 11:**
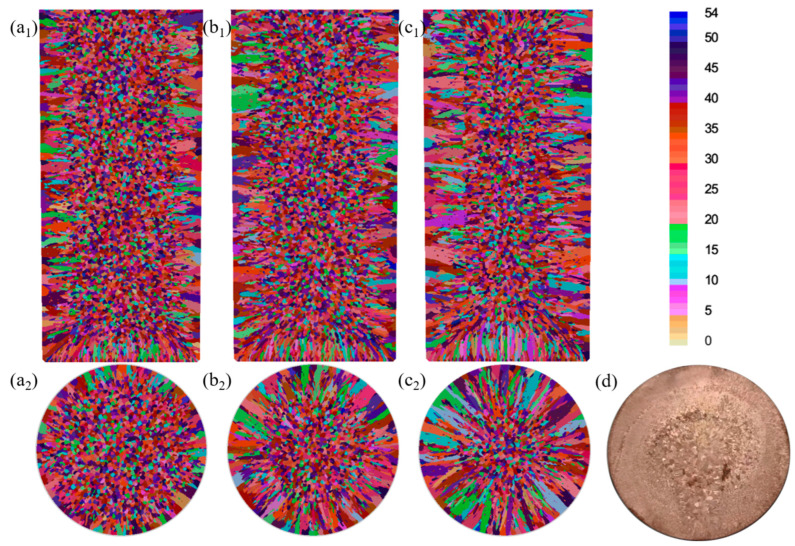
Simulated microstructure of the Cu-3Ti-0.2Fe alloy under different cooling conditions: (**a1**,**a2**) slow cooling; (**b1**,**b2**) air cooling; (**c1**,**c2**) water cooling; (**d**) experimental results (water-cooled).

The numerical simulations in Figure 11a–c demonstrate the cooling-rate-dependent microstructure evolution of the Cu-3Ti-0.2Fe alloy, Table 6 and Table 7 present the simulation statistics results, where increasing heat transfer coefficients from 100 to 5000 W/(m^2^·K) produce three key effects: (1) the progressive expansion of columnar grain zones from 9 mm to over 18 mm from the surface with corresponding equiaxed region reduction, (2) grain coarsening, evidenced by 15–40% decreases in grain counts (longitudinal, 8571 → 7278; cross-section, 2829 → 2015), and (3) 21–40% increases in average grain area (longitudinal, 3.66 → 4.44 × 10^−6^ m^2^; cross-section, 3.59 → 5.03 × 10^−6^ m^2^). These changes confirm that intensified cooling promotes columnar growth while simultaneously reducing nucleation density and enabling grain coalescence. The simulated and experimental results for the solidification structure under water cooling conditions are shown in Figure 11d, where the simulation results, including both columnar and equiaxed crystal regions, match the experimental results quite well. 

The interplay between thermal and flow fields critically governs solidification microstructure development, where the heat transfer coefficient (h) serves as the dominant control parameter. For h = 5000 W/(m^2^·K), three synergistic effects emerge: (1) steep pre-solidification temperature gradients (≥200 K/cm) drive columnar grain elongation and coarsening, (2) suppressed liquidus-front flow velocities (<0.1 m/s) inhibit dendritic fragmentation, and (3) constrained solute transport (Péclet number < 0.5) prevents undercooled zone formation, collectively promoting coarse columnar structures. Conversely, h = 100 W/(m^2^·K) creates opposite conditions: broad solid–liquid zones (>15 mm width) enhance free crystal preservation, while moderate temperature gradients (~50 K/cm) and accelerated flows (>0.3 m/s) jointly facilitate (i) increased nucleation densities (≥105 nuclei/mm^3^), (ii) undercooling amplification (ΔT ≥ 5 K), and (iii) equiaxed grain dominance (>70% area fraction). These dual regimes demonstrate how h quantitatively determines the columnar-to-equiaxed transition through coupled thermosolutal mechanisms.

**Figure 12:**
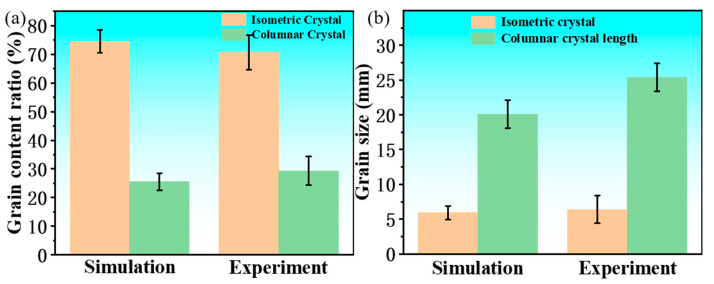
Proportions and sizes of each crystal form: (**a**) proportion; (**b**) size.

To verify the simulation results, they were compared with the experimental results. Based on Nano Measurer software (version 1.2.5, USTC, Hefei, China), the percentages of the crystal regions and the grain sizes corresponding to Figure 5b,d in the simulation and the experiment were statistically analyzed. The results are shown in Figure 12. The measurements showed that the percentage of equiaxed crystals in the experiment was 74.5%, the percentage of columnar crystals was 25.5%, and the average grain size of equiaxed crystals was 5.9 ± 1.2 mm, while the average grain length of columnar crystals was 20.1 ± 1.8 mm. In the simulation, the percentage of equiaxed crystals was 70.7%, the percentage of columnar crystals was 29.3%, the average grain size of equiaxed crystals was 6.4 ± 1.4 mm, and the average grain length of columnar crystals was 25.4 ± 1.9 mm. By comparing these values, it can be seen that the solidification structure of the ingot obtained from the simulation, both in terms of the percentage of equiaxed crystal regions and the grain size, is basically consistent with the experimental results.

#### 3.3.2. Effect of Pouring Temperature on Solidification Microstructure

**Figure 13:**
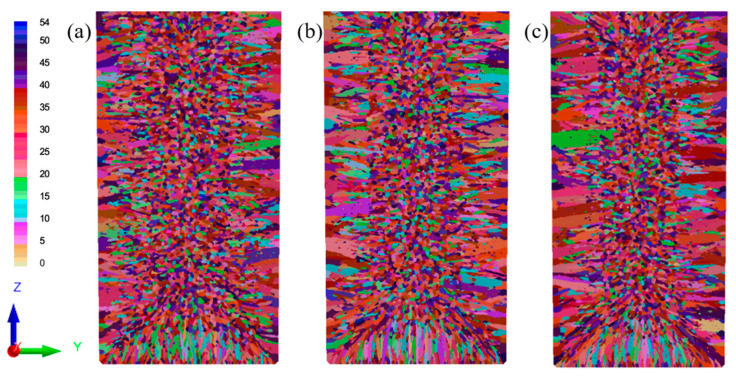
The simulated microstructures of the Cu-3Ti-0.2Fe alloy at various pouring temperatures: (**a**) 1100 °C; (**b**) 1150 °C; (**c**) 1200 °C.

The initial casting temperature fundamentally governs the resultant solidification morphology through its influence on nucleation behavior, crystal growth kinetics, and solute distribution dynamics. High pouring temperatures lead to larger grain sizes and improved flowability but may result in defects and uneven alloy composition. In contrast, low pouring temperatures favor grain refinement but are more prone to defects, with rapid cooling causing thermal stress and deformation. Optimizing pouring temperatures is crucial in controlling metal microstructure and properties. This study investigated the influence of pouring temperatures on solidification behavior through numerical simulations. Figure 13 illustrates the resulting microstructures, while Table 8 quantifies the effects of superheating. The results reveal that higher pouring temperatures promote columnar grain growth, expanding both the width and extent of the columnar zone. This occurs because prolonged cooling at elevated temperatures enhances nucleation and growth kinetics at the solidification front, favoring columnar development over equiaxed grain formation in the central region.

The variation in pouring temperature significantly influences grain morphology through its effects on nucleation and growth dynamics. Elevated pouring temperatures reduce supercooling, which increases the critical nucleation radius while decreasing nucleation rates and weakening inter-nucleation competition, consequently promoting grain coarsening. Conversely, lower pouring temperatures enhance supercooling, generating abundant heterogeneous nucleation sites whose competitive interactions inhibit grain growth and produce refined equiaxed structures. The quantitative analysis of cross-sectional grain characteristics (Figure 13, Table 8) confirms these mechanisms, showing that increasing the pouring temperature from 1100 °C to 1200 °C reduces the grain density while expanding the average grain radius from 0.1665 mm to 0.1820 mm, with corresponding changes in maximum and minimum grain dimensions. The microstructure also shows a gradual decrease in the equiaxed grain fraction of the ingot. Specifically, the cross-sectional area of the largest grain increases from 1.600 cm^2^ to 3.399 cm^2^, while the smallest grains (the tiny grains between columnar crystals) maintain an area of 0.0009 cm^2^, which is essentially unaffected by the temperature change.

## 4. Conclusions

This numerical simulation study systematically examined the solidification behavior of the Cu-3Ti-0.2Fe alloy, comparing three distinct cooling regimes (water cooling, air cooling, and slow cooling) to characterize their effects on thermal profiles, melt flow dynamics, and microstructural evolution. The investigation further evaluated how varying pouring temperatures influence the resulting solidification morphology. The following conclusions were drawn:

(1) Solidification Process: Water cooling leads to the highest solidification rate (2.71 mm/s) but is unstable, while air cooling has a moderate rate (1.45 mm/s), and slow cooling shows the most uniform temperature distribution with the lowest stable rate (0.67–0.95 mm/s).

(2) Alloy Melt Flow: Cooling conditions significantly influence melt flow. Water cooling causes upward fluid motion and potential turbulence, while slow cooling results in surface-directed flow, and air cooling pushes the melt outward from the center.

(3) Heat Transfer and Pouring Temperature: Increasing the heat transfer coefficient (100 to 5000 W/(m^2^·K)) promotes columnar crystal growth and grain coarsening. Higher pouring temperatures (1100 °C to 1200 °C) enhance columnar crystal development, while lower temperatures favor fine equiaxed crystals.

(4) Crystal Proportions: After analysis using Nano Measurer software, the proportion of equiaxed crystals in the experiment was 74.5%, with a size of 5.9 ± 1.2 mm, and the proportion of columnar crystals was 25.5%, with a length of 20.1 ± 1.8 mm. In the simulation, the proportion of equiaxed crystals was 70.7%, with a size of 6.4 ± 1.4 mm, and the proportion of columnar crystals was 29.3%, with a length of 25.4 ± 1.9 mm. The proportion of equiaxed crystals and the grain size were highly consistent in both cases.

The model in this study systematically simulates the Cu-3Ti-0.2Fe alloy solidification process but has limitations. It overlooks factors such as microsegregation, porosity, and surface roughness, which impact component distribution, casting quality, and heat transfer. These factors should be considered in future research to improve model accuracy and practicality.

## Figures and Tables

**Table 1:** Physical parameters of Cu-3Ti-0.2Fe alloy.

Parameter	Value
Thermal conductivity K_T_, (W/(m K))	160.35155
Liquid phase temperature *T_L_*, °C	1050
Solid phase temperature *T_S_*, °C	880
Gibbs Thomson coefficient Γ, m k	3 × 10^7^
Density *ρ*, kg/m^3^	7.65473

**Table 2:** Ingot alloy composition (wt%).

Elements	Cu	Ti	Fe
Cu-3Ti-0.2Fe	96.8	3	0.2

**Table 3:** Mold alloy composition (wt%).

Elements	Cu	Cr	Zr
Cu-Cr-Zr	99.01	0.9	0.09

**Table 4:** Variations in nucleation parameters in the simulation.

Serial Number	Δ*T_v,max_* [k]	Δ*T_v,σ_* [k]	*n_v,max_* [m^−3^]	Δ*T_s,max_* [k]	Δ*T_s,σ_* [k]	*n_s,max_* [m^−2^]
M1	1	0.1	1 × 10^8^	5	0.1	1 × 10^6^
M2	2.5	0.1	1 × 10^8^	5	0.1	1 × 10^6^
M3	5	0.1	1 × 10^8^	5	0.1	1 × 10^6^

**Table 5:** Microstructure simulation parameters.

Microstructure Simulation Parameters	Value
Gibbs–Thomson	3 × 10^−7^
a_2_	1.850831 × 10^−7^
a_3_	2.019982 × 10^−6^
Δ*T_s,max_*	5 K
Δ*T_v,max_*	2.5 K
Δ*T_s,σ_*	0.1 K
Δ*T_v,σ_*	0.1 K
*n_s,max_*	1 × 10^6^ m^−2^
*n_v,max_*	1 × 10^8^ m^−3^

**Table 6:** Statistical results of the simulated longitudinal and transverse sections for the Cu-3Ti-0.2Fe alloy.

Statistical Items	Slow Cooling	Air Cooling	Water Cooling
Nb crystals	8571	8351	7278
Mean surface area/m^2^	3.664 × 10^−6^	3.746 × 10^−6^	4.441 × 10^−6^
Minimum surface area/m^2^	9 × 10^−8^	9 × 10^−8^	9 × 10^−8^
Maximum surface area/m^2^	1.342 × 10^−4^	1.543 × 10^−4^	2.505 × 10^−4^
Mean radius/m	1.532 × 10^−3^	1.590 × 10^−3^	1.731 × 10^−3^

Note: Nb crystals—the number of crystals in the solidified structure; mean surface area—average surface area of the crystals; minimum surface area—surface area of the minimum crystal; maximum surface area—surface area of the largest crystal; mean radius—average radius of the crystals.

**Table 7:** Statistical results of the simulated cross-section for the Cu-3Ti-0.2Fe alloy.

Statistical Items	Slow Cooling	Air Cooling	Water Cooling
Nb crystals	2829	2326	2015
Mean surface area/m^2^	3.586 × 10^−6^	4.361 × 10^−6^	5.034 × 10^−6^
Minimum surface area/m^2^	9 × 10^−8^	9 × 10^−8^	9 × 10^−8^
Maximum surface area/m^2^	8.604 × 10^−5^	1.317 × 10^−4^	1.719 × 10^−4^
Mean radius/m	1.487 × 10^−3^	1.801 × 10^−3^	1.982 × 10^−3^

**Table 8:** Statistical results of the simulated Cu-3Ti-0.2Fe alloy at different pouring temperatures.

Statistical Items	1100 °C	1150 °C	1200 °C
Nb crystals	8063	7278	6666
Mean surface area/m^2^	3.982 × 10^−6^	4.441 × 10^−6^	4.816 × 10^−6^
Minimum surface area/m^2^	9 × 10^−8^	9 × 10^−8^	9 × 10^−8^
Maximum surface area/m^2^	1.600 × 10^−4^	2.505 × 10^−4^	3.399 × 10^−4^
Mean radius/m	1.665 × 10^−3^	1.731 × 10^−3^	1.820 × 10^−3^

## Data Availability

The raw data supporting the conclusions of this article will be made available by the authors on request.
